# Molecular Characterization and Expression Profiles of the Ovine *LHβ* Gene and Its Association with Litter Size in Chinese Indigenous Small-Tailed Han Sheep

**DOI:** 10.3390/ani10030460

**Published:** 2020-03-10

**Authors:** Weimin Wang, Yongfu La, Fadi Li, Shijia Liu, Xiangyu Pan, Chong Li, Xiaoxue Zhang

**Affiliations:** 1 College of Animal Science and Technology, Gansu Agricultural University, Lanzhou 730070, China; wangwm@gsau.edu.cn (W.W.); m15294158469@163.com (Y.L.); lifd@lzu.edu.cn (F.L.); 15604258787@163.com (S.L.); bendanpanxiangyu@163.com (X.P.); lichong@gsau.edu.cn (C.L.); 2 Engineering Laboratory of Sheep Breeding and Reproduction Biotechnology in Gansu Province, Minqin 730020, China

**Keywords:** *LHβ*, genetic characteristic, expression profiles, litter size, sheep

## Abstract

**Simple Summary:**

Litter size is one of the most important reproductive traits in sheep, and the luteinizing hormone beta polypeptide (LHβ) plays an important role in mammalian follicular development. In this study, we cloned and analyzed the cDNA sequence of the ovine *LHβ* gene, and the expression patterns of *LHβ* were determined. Furthermore, the synonymous mutation g.727C > T detected in the *LHβ* gene was confirmed to be significantly associated with litter size (*p* < 0.01). These findings support *LHβ* g.727C > T as a genetic marker for litter size in sheep.

**Abstract:**

The luteinizing hormone beta polypeptide (LHβ) is a glycoprotein hormone secreted by basophilic granular cells of the adenohypophysis, and plays an important role in mammalian follicular development. In this study, we cloned and analyzed the cDNA sequence of the ovine *LHβ* gene. RT-qPCR analysis showed that ovine *LHβ* was widely expressed in tissues, with significantly higher expression in the hypophysis than that in other tissues (heart, liver, spleen, lung, kidney, rumen, duodenum, muscle, fat, hypothalamus, and sex glands) (*p* < 0.01). Hypophyseal expression of *LHβ* mRNA in lamb increased with age and reached a peak at 70 days, although a slight decrease was observed at 84 days of age. In addition, the synonymous mutation g.727C > T detected in the *LHβ* gene was confirmed to be significantly associated with the litter size (*p* < 0.01). Ewes carrying the *TT* genotype produced more lambs than those carrying the *TC* and *CC* genotypes (0.42 and 0.39 per delivery, respectively; *p* < 0.05). Our results confirm the association of ovine *LHβ* with litter size in Small-Tailed Han Sheep and implicate *LHβ* as a candidate for improving reproductive traits in agricultural sheep breeding programs.

## 1. Introduction

The luteinizing hormone (LH) is a glycoprotein hormone secreted by basophilic granular cells of the adenohypophysis [1]. This gonadotropin plays a central role in vertebrate reproductive function by conveying integrated central information from the hypothalamic–pituitary complex to the gonads in both males and females [2]. LH is a heterodimer, comprising a common α subunit (LHα) and a hormone-specific β subunit (LHβ), each encoded by a single gene [3]. The rat [4], human [5], bovine [6], horse [7], chicken [8], and sheep [9,10] forms of the *LHβ* gene have been cloned. Sheep *LHβ* is located on chromosome 14 and comprises three exons separated by two intronic regions [11].

*LHβ* is an important candidate involved in reproduction traits and variants may contribute to some pathologies of pituitary–gonadal function [12]. Nilsson et al. found that the high frequency of the *LH* variants worldwide represents an important confounding factor that leads to disproportionately low *LHβ* levels detected in some immunometric assays [12]. The *LHβ* c.118_120del mutation results in hypogonadism due to intracellular sequestration of the hormone [13].

The Small-Tailed Han Sheep was originally raised in Shandong Province, China [14]. It quickly gained the attention of Chinese sheep breeders and is commonly used as the female parent in modern hybridization systems because of its reputation for high fertility [15]. The objectives of the present study were: (1) to clone and characterize the cDNA sequence of the ovine *LHβ* gene; (2) to analyze the expression profiles of ovine *LHβ* m RNA in different tissues in rams and ewes; (3) to investigate the association of polymorphisms in the ovine *LHβ* gene with litter size in Small-Tailed Han Sheep. This study could provide a valuable molecular marker for marker-assisted selection (MAS) in Small-Tailed Han Sheep breeding programs.

## 2. Materials and Methods

### 2.1. Ethics Statement

All experiments in this study were carried out in accordance with the approved guidelines from the Regulation of the Standing Committee of Gansu People’s Congress under Permission No. 2019-00014. All experimental protocols and the collection of samples were approved by the Ethics Committee of Gansu Agriculture University (China) under Permission No. 2002-58.

### 2.2. cDNA Cloning and Sequence Analysis

The sheep *LHβ* cDNA sequence (GenBank Accession no. NM_001009380.1) was employed as a template. Primer pairs were designed based on the whole genome and coding sequence region of the *LHβ* gene (Table 1). The functional domains of the deduced proteins were analyzed with the Simple Modular Architecture Research Tool (SMART) (http://smart.embl-heidelberg.de/) [16]. The homologies of *LHβ* coding products in different species were analyzed by ClustalW2 online (http://www.ebi.ac.uk/Tools/msa/clustalw2/).

The RNA used in this process was extracted from the tissues of an adult female Chinese indigenous Small-Tailed Han Sheep (Gansu Province, China) with TransZol (TransGen Biotech, Beijing, China). RT-PCR (reverse transcription PCR) was performed by using Taq polymerase (TransGen Biotech, Beijing, China) and TransScript One-Step gDNA Removal and cDNA Synthesis SuperMix (TransGen Biotech, Beijing, China). Following separation by agarose gel electrophoresis, the predominant PCR product was purified and subsequently cloned into the pEGM-T-Easy vector (Promega, WI, USA) prior to sequencing.

### 2.3. Analysis of Spatio-Temporal Expression of LHβ in Sheep

Sheep *LHβ* mRNA levels were detected in different tissues (heart, liver, spleen, lung, kidney, rumen, duodenum, muscle, fat, hypothalamus, hypophysis, sex glands (testicle and ovary)) of six Small-Tailed Han Sheep (three adult rams and three adult ewes). The sheep *LHβ* mRNA levels in the hypophysis were quantified at different ages by quantitative real-time PCR (qPCR). Forty-two lambs were slaughtered for hypophysis tissue sampling at 0 d, 14 d, 28 d, 42 d, 56 d, 70 d, and 84 d (six lambs were slaughtered at each time-point). The total RNA of each tissue was extracted and subsequently reverse-transcribed into cDNA, as described previously. A 60 bp fragment of sheep *LHβ* was amplified by PCR using specific primers (*LHβ*-expression-S, *LHβ*-expression-A for *LHβ*, Table 1) and the following thermocycling conditions: 3 min at 94 °C, followed by 30 s at 94 °C, 30 s at 65.1 °C, and 30 s at 72 °C (34 cycles), and a final extension incubation at 5 min at 72 °C. GAPDH was used as an internal control gene. qPCR was performed on the BIO-RAD CFX96 Touch Real-time PCR Detection System (BIO-RAD, USA) using SYBR Green Real-time PCR Master Mix (TOYOBOCO, Osaka, Japan) as the readout. Data were analyzed using the 2^−^^△△CT^ method [17].

### 2.4. SNP Identification and Association Analysis

A total of 869 ewes (aged 12–30 months) were obtained from Minqin Zhongtian Sheep Ltd. (Wuwei, China). All the sheep were produced using the artificial insemination system and raised under the same management conditions. The rams that were mated with these ewes had been sold, and blood samples were not collected for genotyping. All ewes lambed between July and August 2015. The litter size data for ewes was recorded for the first or second parity. The DNA used in this process was extracted from the blood of 869 Small-Tailed Han Sheep [15]. The SNP of the ovine *LHβ* gene was identified by sequencing the PCR products amplified using the eight mixed DNA samples obtained from Small-Tailed Han Sheep blood samples. Specific primers for use in the polymerase chain reaction-restriction fragment length polymorphism (PCR-RFLP) method were designed based on the assembled DNA sequence of the sheep *LHβ* gene (Table 1). The PCR for genotyping was performed in a volume of 25 μL, containing 10× PCR buffer, 0.35 μM primer, 87.5 μM dNTPs, 50 ng genomic DNA, and 1.25 U Taq DNA Polymerase (TransGen Biotech) using the following thermocycling conditions: 5 min at 94 °C, followed by 30 s at 94 °C, 30 s at 60 °C, 30 s at 72 °C (35 cycles), and a final extension incubation for 5 min at 72 °C. Samples of the PCR product (4 µL) were digested with 2 U *BsrB* I for 30 min at 37 °C and then separated by agarose gel (3%) electrophoresis stained with GelRed.

The PROC GLM procedure in SAS software package was employed to analyze the association between genotypes and trait [18] using a linear model with the fixed effects:

Y_ij_ = μ + G_i_ + P_j_ + ε_ij_ (1)

where Y_ij_ represents the i traits observation value, μ represents the mean; G_i_ represents the effect of the ith genotypes, P_j_ represents the effect of the jth parity (j = 1, 2), and ε_ij_ represents the residual corresponding to the traits observation value with variance. G_i_ and P_j_ were fixed effects. *p* < 0.05 was considered to indicate statistical significance.

## 3. Results

### 3.1. Sequence Analysis of Ovine LHβ

**Figure 1:**
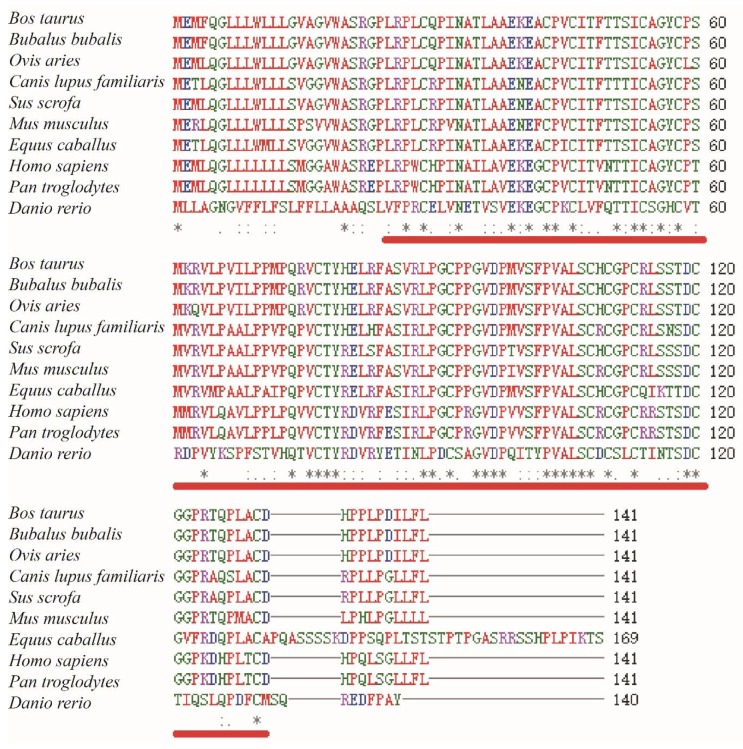
Multiple amino acid sequence alignments of *Bos taurus* (NM_173930.1), *Bubalus bubalis* (NM_001290957.1), *Ovis aries* (NM_001009380.1), *Canis lupus familiaris* (NM_001197033.1), *Sus scrofa* (XM_005664701.1), *Mus musculus* (NM_008497.2), *Equus caballus* (NM_001197093.1), *Homo sapiens* (NM_000894.2), *Pan troglodytes* (NM_001071803.1), and *Danio rerio* (NM_205622.2). Glycoprotein hormone beta chain (GHβ) homologues are indicated by red underlining. The same sequence is indicated by an asterisk. The conservative and semi-conservative mutations are represented by “:” and “.”, respectively.

A 1018 bp fragment of the ovine LHβ gene was cloned. The ovine LHβ gene contained a putative open reading frame (ORF) of 426 bp, encoding a protein consisting of 141 amino acid residues. The GHβ domain was predicted at the N-terminal of the LHβ protein sequence. Multiple amino acid sequence alignments of cattle, buffalo, sheep, dog, pig, rat, horse, human, chimpanzee, and zebrafish LHβ proteins revealed a high degree of conservation among LHβ proteins in different species (Figure 1).

### 3.2. Analysis of Spatio-Temporal Expression of LHβ in Sheep

**Figure 2:**
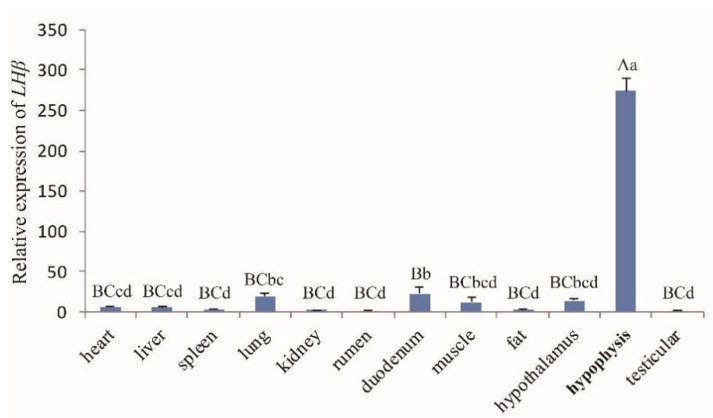
The *LHβ* mRNA expression profile of various tissues in Hu rams. Note: Different capital letters indicate an extremely significant difference (*p* < 0.01), and different lowercase letters indicate a significant difference (*p* < 0.05).

**Figure 3:**
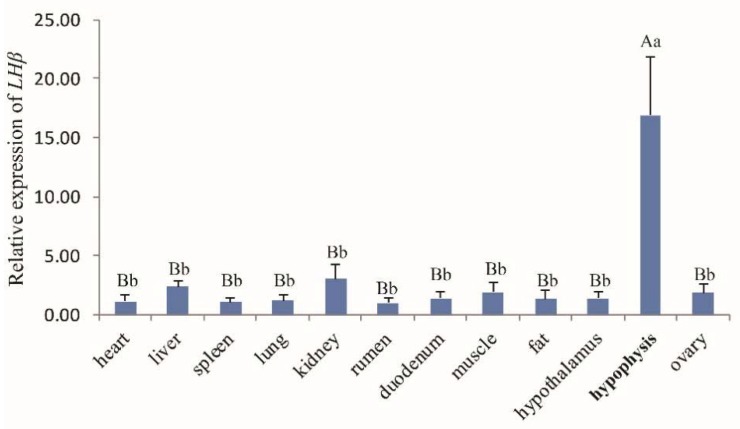
The *LHβ* mRNA expression profile of various tissues in Hu ewes. Note: Different capital letters indicate an extremely significant difference (*p* < 0.01), and different lowercase letters indicate a significant difference (*p* < 0.05).

The general tissue distribution of the LHβ gene in rams and ewes was investigated by real-time PCR (Figure 2 and Figure 3, respectively). LHβ expression was found to be ubiquitous and was detected in all 13 tissues tested (heart, liver, spleen, lung, kidney, rumen, duodenum, muscle, fat, hypothalamus, hypophysis, testis (Figure 2), and ovary tissues (Figure 3), and the LHβ gene displayed significantly higher expression in hypophysis than in other tissues (*p* < 0.01).

**Figure 4:**
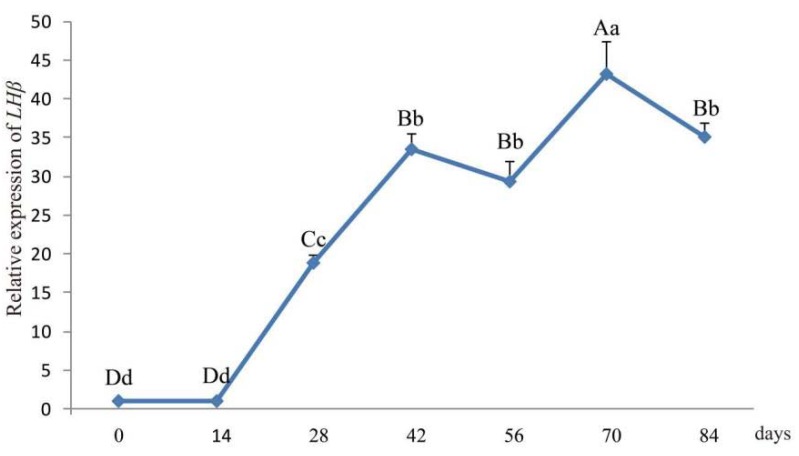
Developmental changes in hypophyseal *LHβ* mRNA expression in lambs aged 0–84 days. Note: Different capital letters indicate an extremely significant differences (*p* < 0.01), and different lowercase letters indicate a significant difference (*p* < 0.05).

The *LHβ* mRNA expression levels in ram hypophysis were quantified by real-time PCR at seven different time-points (0 d, 14 d, 28 d, 42 d, 56 d, 70 d, and 84 d; Figure 4). The expression level of *LHβ* mRNA in ram hypophysis increased with age and reached a peak at 70 d followed by a slight decrease at 84 d.

### 3.3. SNP Scanning of Ovine LHβ Gene

**Figure 5:**
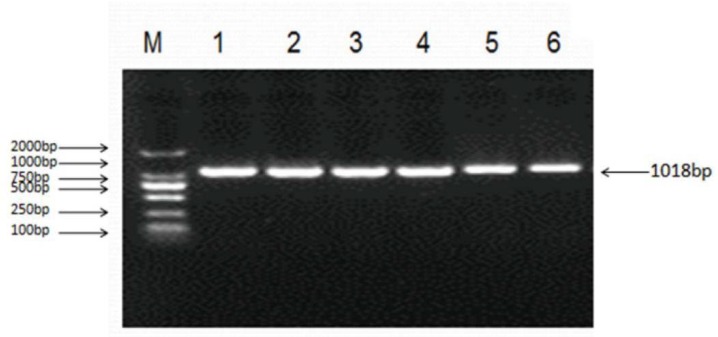
Polymerase chain reaction (PCR) amplification of the ovine *LHβ* gene. 1~6 Lane: 1018 bp fragment of ovine LHβ gene; M: DNA Marker (DL2000).

**Figure 6:**
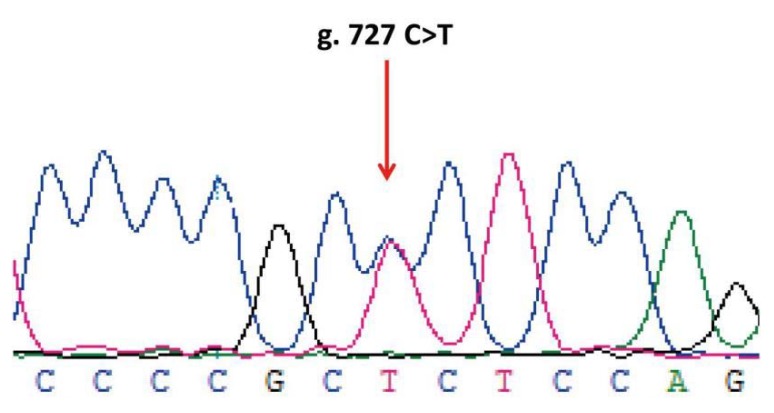
Sequencing results of the ovine *LHβ* gene. The red arrow indicates the mutant peaks.

**Figure 7:**
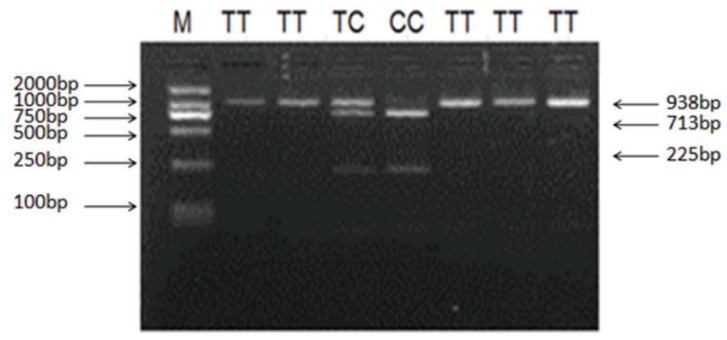
PCR-Restriction fragment length polymorphism (PCR-RFLP) results of different genotypes of the PCR products digested by enzyme BsrB I g.727C > T of ovine *LHβ* polymorphisms. The genotypes are marked at the top of the lanes. M: DNA Marker (DL2000).

Sequencing of 1018 bp PCR product was amplified from a DNA pool obtained from eight Small-Tailed Han Sheep using the primer pair LHβ-SNP-S and LHβ-SNP-A (Table 1, Figure 5) revealed a synonymous mutation, g.727C > T in the sheep LHβ gene (Figure 6). This mutation was confirmed by PCR-RFLP analysis using the BsrB I restriction enzyme, which generated a 938 bp band representing the allele C, and the bands of 713 bp and 225 bp representing the allele T (Figure 7).

### 3.4. Association Analysis of Ovine LHβ Gene with the Litter Size

The effect of SNP in the ovine LHβ gene on the litter size in the Small-Tailed Han Sheep was studied. Association analysis results indicated that the g.727C > T polymorphism of the ovine LHβ gene was significantly associated with the litter size (*p* < 0.05) (Table 2). Moreover, the litter sizes produced by ewes carrying the TT genotype were increased compared with those produced by the ewes carrying CC and TC genotypes (0.39 and 0.42 per delivery, respectively).

## 4. Discussion

In this study, the GHβ domain was predicted to be formed by amino acid residues 25–131 of the LHβ protein by the SMART programme. Glycoprotein hormones (or gonadotropins) are a family of proteins that include the follicle-stimulating hormone (FSH), LH, thyrotropin (TSH), and chorionic gonadotropin (CG), as well as at least two forms of fish gonadotropin [19]. All these hormones consist of two glycosylated chains (alpha and beta) [20]. In mammalian gonadotropins, the alpha chain is identical in the four types of hormones, while the beta chains, despite being homologous, are different [21]. Multiple amino acid sequence alignments of sheep, cattle, human, mouse, pig, zebrafish, dogs, horse, buffalo, and chimpanzee LHβ proteins conducted in this study showed a high degree of conservation among the LHβ proteins in different species (Figure 1). Furthermore, this amino acid sequence alignment showed that the GHβ domain is highly conservative among the predicted sheep LHβ protein and other species, indicating a high level of functional similarity among the GHβ domains in different species. These findings verify that the sheep LHβ gene encodes a glycoprotein hormone.

The tissue expression profiles obtained by RT-qPCR analysis revealed the broad expression pattern of the LHβ gene in Small-Tailed Han Sheep, with expression detected in all 13 of the tissues tested (heart, liver, spleen, lung, kidney, rumen, duodenum, muscle, fat, hypothalamus, hypophysis, and sexual glands (testicle and ovary). The LHβ gene was expressed at very high levels in the hypophysis (*p* < 0.01), which is consistent with the secretion of LH by basophilic granular cells in the adenohypophysis [1]. It should be noted that the expression of the LHβ gene in the hypophysis of rams is significantly higher than that in ewes, suggesting that the effect of the LHβ gene on hypophysis in rams may be greater than that in ewes. However, further experiments are needed to verify this hypothesis. The potential function of the ovine LHβ gene during the development of the ovine hypophysis was investigated by RT-qPCR analysis of LHβ mRNA expression in the lambs aged 0–84 days. Hypophyseal expression of LHβ mRNA in rams increased with age, reaching a peak at 70 d. Pulsatile secretion of LH has been reported to play a key role in the onset of puberty in mammals, including sheep [22,23]. Small-Tailed Han Sheep is a breed recognized for its high fecundity and precocity, with puberty occurring between 2 and 4 months of age. Therefore, we concluded that the male Small-Tailed Han lamb reproductive apparatus matures gradually with age, and that puberty occurs at around 70 days of age. Furthermore, our analysis indicates a relationship between the expression of LHβ mRNA and puberty in lambs, although further study is needed and the mechanisms remain unclear.

Previous studies related to LHβ gene polymorphisms have focused mainly on human reproductive disorders [24,25]. Basavarajappa et al. identified seven different variants of the LHβ gene in the Indian river buffalo (*Bubalus bubalis*) based on a polymerase chain reaction-single-strand conformation polymorphism (PCR-SSCP) analysis [26]. Li et al. detected a g.401G > A/C mutation in the caprine LHβ gene that was significantly associated with litter size (*p* < 0.05) in Nanjiang-Huang goats [27]. Additionally, Liu et al. also found six synonymous mutations in the 5ʹ UTR and exon 2 of the ovine LHβ gene, two of which were significantly associated with litter size (*p* < 0.01) in Hu sheep [28]. The g.727C > T mutation of the LHβ gene identified in this study is also a synonymous mutation. Nonetheless, recent studies suggest synonymous mutations can change protein levels or protein conformation by altering regulatory splice sites, mRNA stability, miRNA binding sites, or translation efficiency [29]. To further investigate the effect of this synonymous mutation on sheep phenotype, we analyzed the potential association between genotype and litter size. The results indicated that the novel SNP g.727C > T mutation in the ovine LHβ gene was significantly associated to the trait of litter size. The phenotype values of the litter size in the animals with the TT genotype were evidently higher than those with the TC and CC genotypes. Furthermore, the litter sizes produced by ewes carrying the TT genotype were increased compared with those produced by the ewes carrying TC and CC genotypes (0.42 and 0.39 per delivery, respectively). Since the luteinizing hormone is the coding product of the LHβ gene and it can promote ovulation, we speculate that the mutation may affect the ovulation number of ewes by regulating the secretion level of the luteinizing hormone. Nonetheless, further studies are needed to verify the physiological functions of the LHβ gene in high prolificacy at the cell and protein levels.

## 5. Conclusions

Our results indicate the potential of the ovine *LHβ* gene as a candidate for improving litter size in the agricultural sheep breeding program. Further studies on the association between the *LHβ* gene and reproductive performance are warranted in larger populations of different breeds and under different environmental conditions to confirm these results.

## Figures and Tables

**Table 1:** Primer pairs designed for the sheep *LHβ* and *GAPDH* genes.

Primer Name	GenBank Accession Number	Primer Sequences (5ʹ–3ʹ)	Annealing Temperature (°C)	Product Size (bp)
*LHβ*–SNP-S	NC_019471.1	TGCTCCAGGTAAGTCTGTAGGG	65.7	1018 bp
*LHβ*–SNP-A		AGCGTCTGCTGGCTTTGG		
*LHβ*-expression-S	NM_001009380.1	ACCCTGGCGGCTGAGAA	60	60 bp
*LHβ*-expression-A		GCAGATGCTGGTGGTGAAAG		
*GAPDH*-S	NM_001190390.1	ACTTTGGCATCGTGGAGG	58	379 bp
*GAPDH*-A		GAAGAGTGAGTGTCGCTGTTG		

**Table 2:** Association between genotypes carrying the g.727C > T polymorphism of the ovine *LHβ* gene and litter size.

Genotypes	Number of Animals	Litter Size
*CC*	637	1.78 ± 0.02 ^b^
*TC*	168	1.75 ± 0.05 ^b^
*TT*	84	2.17 ± 0.08 ^a^

Phenotype values are presented as mean ± standard error. Phenotype values in the same column with different superscript lowercase indicate extremely significant differences (*p* < 0.05).

## References

[1] Gharib S.D., Wierman M.E., Shupnik M.A., Chin W.W. (1990). Molecular biology of the pituitary gonadotropins. Endocr. Rev..

[2] Long H.M.E.A. (1922). Characteristic effects upon growth, oestrus and ovulation induced by the intraperitoneal administration of fresh anterior hypophyseal substance. Proc. Natl. Acad. Sci. USA.

[3] Li M. (1998). A comprehensive evolutionary analysis based on nucleotide and amino acid sequences of the alpha- and beta-subunits of glycoprotein hormone gene family. J. Endocrinol..

[4] Jameson L., Chin W.W., Hollenberg A.N., Chang A.S., Habener J.F. (1984). The gene encoding the beta-subunit of rat luteinizing hormone. Analysis of gene structure and evolution of nucleotide sequence. J. Biol. Chem..

[5] Talmadge K., Vamvakopoulos N.C., Fiddes J.C. (1984). Evolution of the genes for the p-subunit of human chorionic gonadotropin and luteinizing hormone. Nature.

[6] Virgin J.B., Silver B.J., Thomason A.R., Nilson J.H. (1985). The gene for the beta subunit of bovine luteinizing hormone encodes a gonadotropin mrna with an unusually short 5′-untranslated region. J. Biol. Chem..

[7] Sherman G.B., Wolfe M.W., Farmerie T.A., Clay C.M., Threadgill D.S., Sharp D.C., Nilson J.H. (1992). A single gene encodes the beta-subunits of equine luteinizing hormone and chorionic gonadotropin. Mol. Endocrinol..

[8] Noce T., Ando H., Ueda T., Kubokawa K., Higashinakagawa T., Ishii S. (1989). Molecular cloning and nucleotide sequence analysis of the putative cdna for the precursor molecule of the chicken lh-β Subunit. J. Mol. Endocrinol..

[9] Mountford P.S., Bello P.A., Brandon M.R., Adams T.E. (1989). Cloning and DNA sequence analysis of the cdna for the precursor of ovine follicle stimulating hormone beta-subunit. Nucleic Acids Res..

[10] Gisèle D.A.B., Mohieddine M., Marian J., Raymond C. (1990). Cloning and sequence analysis of the cdna for the precursor of the beta subunit of ovine luteinizing hormone. Nucleic Acids Res..

[11] Faraut T., Kijas J.W., Maddox J.F., Mcewan J.C., Oddy V.H., Raadsma H.W., Wade C., Wang J., Wang W., Xun X. (2010). The sheep genome reference sequence: A work in progress. Anim. Genet..

[12] Nilsson C., Pettersson K., Millar R.P., Coerver K.A., Matzuk M.M., Huhtaniemi I.T. (1997). Worldwide frequency of a common genetic variant of luteinizing hormone: An international collaborative research. Fertil. Steril..

[13] Potorac I., RiveroMuller A., Trehan A., Kielbus M., Jozwiak K., Pralong F., Hafidi A., Thiry A., Menage J., Huhtaniemi I.T. (2016). A vital region for human glycoprotein hormone trafficking revealed by an lhb mutation. J. Endocrinol..

[14] Zhao Y. (2003). Zhong Guo Yang Yang Xue.

[15] Wang W., Liu S., Li F., Pan X., Li C., Zhang X., Ma Y., La Y., Xi R., Li T. (2015). Polymorphisms of the ovine bmpr-ib, bmp-15 and fshr and their associations with litter size in two chinese indigenous sheep breeds. Int. J. Mol. Sci..

[16] Letunic I., Doerks T., Bork P. (2015). Smart: Recent updates, new developments and status in 2015. Nucleic Acids Res..

[17] Livak K., Schmittgen T. (2000). Analysis of relative gene expression data using real-time quantitative pcr and the 2^−^^ΔΔct^ method. Methods.

[18] Pan X., Liu S., Li F., Wang W., Li C., Ma Y., Li T. (2014). Molecular characterization, expression profiles of the ovinefshrgene and its association with litter size. Mol. Biol. Rep..

[19] Stockell Hartree A., Renwick A.G.C. (1992). Molecular structures of glycoprotein hormones and functions of their carbohydrate components. Biochem. J..

[20] Cahoreau C., Klett D., Combarnous Y. (2015). Structure–function relationships of glycoprotein hormones and their subunits’ ancestors. Front. Endocrinol..

[21] Fernández-Tejada A., Vadola P.A., Danishefsky S.J. (2014). Chemical synthesis of the β-subunit of human luteinizing (hlh) and chorionic gonadotropin (hcg) glycoprotein hormones. J. Am. Chem. Soc..

[22] Pelletier J., Carrez-Camous S., Thiery J.C. (1981). Basic neuroendocrine events before puberty in cattle, sheep and pigs. J. Reprod. Fertil..

[23] Nestor C.C., Briscoe A.M.S., Davis S.M., Valent M., Goodman R.L., Hileman S.M. (2012). Evidence of a role for kisspeptin and neurokinin b in puberty of female sheep. Endocrinology.

[24] Liaqat I., Jahan N., Krikun G., Taylor H.S. (2014). Genetic polymorphisms in pakistani women with polycystic ovary syndrome. Reprod. Sci..

[25] Cowan M., Davie A., Migaud H. (2012). Photoperiod effects on the expression of kisspeptin and gonadotropin genes in atlantic cod, gadus morhua, during first maturation. Comp. Biochem. Physiol. Part A Mol. Integr. Physiol..

[26] Basavarajappa M.S., De S., Thakur M., Datta T.K., Dogra G., Yadav P., Goswami S.L. (2008). Characterization of the luteinizing hormone beta (lh-β) subunit gene in the indian river buffalo (bubalus bubalis). Gen. Comp. Endocrinol..

[27] Li L.I., Zhang H.P., Deng-Jun W.U. (2006). Relationship between polymorphism of lhβ gene and reproductive performance of nanjiang-huang goat. Anim. Husb. Vet. Med..

[28] Liu Y., Ying S.J., Wu F.R., Wang F., Wang Z.Y., Zhu T.G., Shi G.Q. (2010). Polymorphism of lhβ gene and its correlation with prolificacy of hu sheep. Jiangsu J. Agric. Sci..

[29] Sharma Y., Miladi M., Dukare S., Boulay K., Caudron-Herger M., Groß M., Backofen R., Diederichs S. (2019). A pan-cancer analysis of synonymous mutations. Nat. Commun..

